# Preparation and Performance of Regenerated Al_2_O_3_-Coated Cathode Material LiNi_0.8_Co_0.15_Al_0.05_O_2_ from Spent Power Lithium-Ion Batteries

**DOI:** 10.3390/molecules28135165

**Published:** 2023-07-02

**Authors:** Liwen Ma, Guangyun Liu, Yuehua Wang, Xiaoli Xi

**Affiliations:** 1 Collaborative Innovation Center of Capital Resource-Recycling Material Technology, Faculty of Materials and Manufacturing, Beijing University of Technology, Beijing 100124, China; maliwen@bjut.edu.cn (L.M.); liuguangyun@emails.bjut.edu.cn (G.L.); 2 National Engineering Laboratory for Industrial Big-Data Application Technology, Beijing University of Technology, Beijing 100124, China; 3 Key Laboratory of Advanced Functional Materials, Ministry of Education, Faculty of Materials and Manufacturing, Beijing University of Technology, Beijing 100124, China; wangyaohua@emails.bjut.edu.cn

**Keywords:** spent power lithium-ion battery, solid phase sintering, regeneration, coated cathode materials

## Abstract

In this study, LiNi_0.8_Co_0.15_Al_0.05_O_2_@x%Al_2_O_3-_coated cathode materials were regeneratively compounded by the solid-phase sintering method, and their structural characterization and electrochemical performance were systematically analyzed. The regenerated ternary cathode material precursor synthesized by the co-precipitation method was roasted with lithium carbonate at a molar ratio of 1:1.1, and then completely mixed with different contents of aluminum hydroxide. The combined materials were then sintered at 800 °C for 15 h to obtain the regenerated coated cathode material, LiNi_0.8_Co_0.15_Al_0.05_O_2_@x%Al_2_O_3_. The thermogravimetry analysis, phase composition, morphological characteristics, and other tests show that when the added content of aluminum hydroxide is 3%, the regenerated cathode material, LiNi_0.8_Co_0.15_Al_0.05_O_2_@1.5%Al_2_O_3,_ exhibits the highest-order layered structure with Al_2_O_3_ coating. This material can better inhibit the production of Ni^2+^, and improve material structure and electrochemical properties. The first charge–discharge efficiency of the battery assembled with this regenerated cathode material is 97.4%, a 50-cycle capacity retention is 93.4%, and a 100-cycle capacity retention is 87.6%. The first charge–discharge efficiency is far better than that of the uncoated regenerated battery.

## 1. Introduction

With the gradual rise of new energy vehicles, the installed capacity of lithium-ion batteries (LIBs) is increasing exponentially, and new LIBs will be scrapped after 5–8 years of use. Noteworthily, 355,000 tons of LIBs were decommissioned in 2019, and it is expected that the retirement of LIBs will reach 800,000 tons by 2025 [1,2,3]. The composition of LIBs is complex, and it is necessary to regenerate them resourcefully. Reducing, reusing, and recycling spent lithium is an important consideration for building a circular economy, and the 3R technology for spent LIBs has become a hot research direction [4]. The spent power battery contains a variety of harmful pollutants and valuable metal elements; therefore, if it is not treated effectively, it will not only cause serious environmental pollution, but also induce a tremendous waste of valuable resources. Thus, the recycling of spent LIBs is in line with China’s sustainable development strategy [5].

At present, the recycling of spent power batteries mainly focuses on the recycling of positive and negative collector fluid, positive electrode material, and negative electrode material. While the positive electrode material is rich in cobalt, nickel, manganese, and other metal elements, its recycling value is much higher than the other two materials. Many researchers have conducted in-depth research on it. The recycling methods for the spent power battery mainly include pyrometallurgy, hydrometallurgy, and microbial metallurgy [6,7,8,9,10]. The main objective is to decompose and extract metal elements such as cobalt (Co), nickel (Ni), and lithium (Li) from spent power batteries by high-temperature roasting, chemical treatment, or microbial effect for the selective recovery of valuable compounds and metals. However, selective metal recovery is a challenging task in terms of the special properties of spent batteries, the requirements for metal purity recovery, and ensuring the sustainability of the recovery process [11].

To date, many mature recycling processes have been employed to manage spent LIBs for obtaining a variety of products such as aluminum (Al), copper (Cu), Co, Ni, Co_2_O_3_, CoCl_2_, and so on, as summarized in Table 1. Moreover, some improved processes have also been explored. For instance, Wu et al. used H_2_SO_4_ as a leaching agent to treat spent LiFePO_4_ batteries and reported that 97% of Li could be leached into the solution while more than 99% of Fe remained in the residue [12].Chen et al. prepared Li_2_CO_3_ from spent LiFePO_4_ by a hydrometallurgical process. The efficiency of extracting high-purity Li^+^ with low impurities in solution was achieved under the optimized extraction conditions (c = 0.4 mol L^−1^, L/S = 7.93 mL g^−1^, = 0.13, and T = 60 °C) [13]. Bahaloo et al. found that citric acid had a facilitating effect on the leaching and recovery of Co, Li, and Mn by Aspergillus niger from spent LIBs. Their recovery rates could reach 100%, 95%, and 70%, respectively [14]. Biswal et al. used the Aspergillus niger MM1 strain to recover Co from fungal extract with Na_2_S, NaOH, and Na_2_C_2_O_4_, and then recovered Li with sodium carbonate. The dissolution rate of Co was more than 82%, and that of Li was more than 100% [15]. JX Nippon Mining & Metals Corporation subsidiary roasted the shell, connector, and wire; recovered the active cathode material; and carried out leaching, solvent extraction, and electrolytic deposition of the cathode material to obtain Ni^3+^ and Co^2+^ as well as Li_2_CO_3_ and MnCO_3_ [16]. Dang et al. converted insoluble Li from spent LIBs into soluble Li by roasting with calcium chloride and recovered Li by up to 90.58% [17]. Wang et al. synthesized H_1.6_Mn_1.6_O_4_ lithium-ion screen adsorbents and applied them to recover metal Li and Co from spent cathode materials. In the citrate-hydrogen peroxide system, the leaching rates of Co and Li were 86.21% and 96.9%, respectively; in the tartaric acid system, the leaching rates of Co and Li were 90.34% and 92.47%, respectively [18].

In addition to obtaining metal salts and other products from spent power batteries, the direct regeneration of spent battery materials has become a research hotspot. In other words, instead of separating the valuable elements, the impurities are removed, and re-synthesis is carried out to obtain regenerated battery materials by liquid-phase or solid-phase preparation processes [22,23]. For example, Tang et al. regenerated LiNi_0.5_Co_0.2_Mn_0.3_O_2_ cathode material by two-stage sintering after supplementing Li_2_CO_3_. The regenerated cathode material showed a first discharge specific capacity of 154.87 mA·h g^−1^ and a capacity retention of 90% after 100 cycles between 2.75–4.2 V [24]. The vanadium doping process improved the conductivity of the recycled cathode material, LiFePO_4_/C. After 200 cycles at 1C, the discharge capacity was 134.3 mA·h g^−1^, and the discharge capacity retention was 99.1% [25]. Ye et al. employed the sulfuration method to recycle the spent LiCoO_2_ cathode material into Li_2_SO_4_ regenerated material at a current density of 2 A g^−1^ for 500 cycles, which exhibited good Na storage performance (500 mA·h g^−1^) [26]. Fan et al. directly regenerated LiNi_0.5_Co_0.2_Mn_0.3_O_2_ cathode material by a series of treatment processes (granulation, ion doping, and heat treatment), and the regenerated material exhibited excellent electrochemical performance with a discharge capacity of 189.8 mA·h g^−1^ at room temperature and for 300 cycles at 1C with the capacity retention of 83.2% [27]. Tang et al. used the traditional regeneration method (solid phase calcination) and the new process (annealing after hydrothermal treatment at 150C) to supplement lithium ion to the spent cathode material in order to obtain the recycled cathode material LiFePO_4_, and the initial capacity of the assembled battery could reach 144.02 mA·h^−1^ [28].

Among cathode materials used for power LIBs, layered Ni-enriched ternary oxides have been widely studied due to their high specific capacity, elevated energy density, non-toxicity, and relatively low cost. LiNi_0.8_Co_0.15_Al_0.05_O_2_ is a promising cathode material for power vehicles. It has also become a popular recycled product because it contains valuable elements such as Co, Ni, Al, and so on. However, our previous study showed that the charge–discharge efficiency of regenerated LiNi_0.8_Co_0.15_Al_0.05_O_2_ batteries was not very high [29]. This may be attributed to the presence of trace impurity ions in the structure of the regenerated cathode material, which hinder the intercalation and deintercalation of Li^+^ and reduce its charge–discharge efficiency. Wang et al. found that when lithium manganate was coated with Al_2_O_3_, the as-formed Al_2_O_3_ layer not only prevented the electrolyte from directly contacting and dissolving lithium manganate but also formed a composite of Li-Al-O as a fast channel for ionic conduction, which improved the electronic and ionic conductivity of the material, thus improving its electrochemical performance [30]. Therefore, it is considered necessary to coat a layer of Al_2_O_3_ on the surface of the regenerated cathode material to improve its charge–discharge efficiency.

In this study, LiNi_0.8_Co_0.15_Al_0.05_O_2_@x%Al_2_O_3_ cathode materials with different contents of the Al_2_O_3_ coating layer were regenerated, the preparation parameters were optimized, and the best regenerated coating cathode material, LiNi_0.8_Co_0.15_Al_0.05_O_2_@1.5%Al_2_O_3_ was obtained. The coated cathode materials were characterized by different methods, including quantitative analysis of composition by X-ray diffractometer (XRD), micromorphology analysis by scanning electron microscope (SEM), ultrastructure analysis by transmission electron microscope (TEM), molecular structure and chemical composition analysis by infrared spectrometer(IR), and sample surface elemental composition and chemical state by X-ray photoelectron spectroscopy(XPS). Finally, the electrochemical performance of the regenerated cathode materials was also evaluated.

## 2. Results and Discussion

### 2.1. Characterization and Analyses

#### 2.1.1. TG Analyses

**Figure 1:**
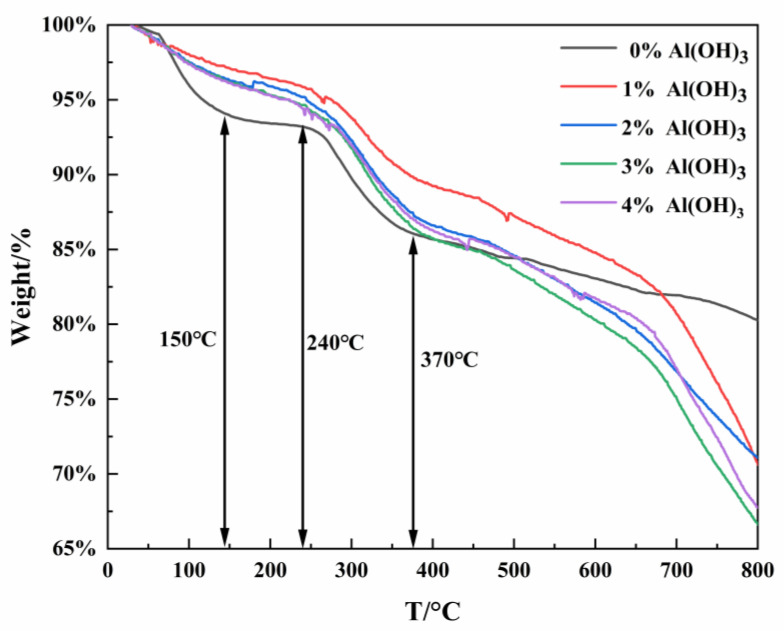
TG results of regenerated cathode materials with different contents of added Al(OH)_3_.

Thermogravimetry (TG) tests were conducted to study the synthesis reaction process of the raw materials used for regenerated coated cathode materials and to research the effect of different added contents of Al(OH)_3_ on the regenerated process of the cathode material. Figure 1 shows the TG curves of mixtures of precursor (The cobalt and nickel hydroxide mixture obtained by coprecipitation after copper removal from the leaching solution of spent power battery cathode materials [29]) and lithium carbonate(Li_2_CO_3_) with different contents of Al(OH)_3_. The results show that the TG curve of the sample without Al(OH)_3_ exhibits a smaller slope than that with Al(OH)_3_ at temperatures between 145 °C and 240 °C, which is attributed to the fact that the added Al(OH)_3_ gets decomposed into Al_2_O_3_ and water, thus leading to an increase in its slope. The overall trend indicates that the slope increased with the increase of added content of Al(OH)_3_ beyond 240 °C, and the slope of the curve was the largest when the addition was 3% or 4%, indicating that the Al(OH)_3_ could promote the reaction and lead to the complete reaction of the Li_2_CO_3_. The promotion effect was the most obvious when the addition of Al(OH)_3_ was 3% or 4%.

#### 2.1.2. XRD Analyses

**Figure 2:**
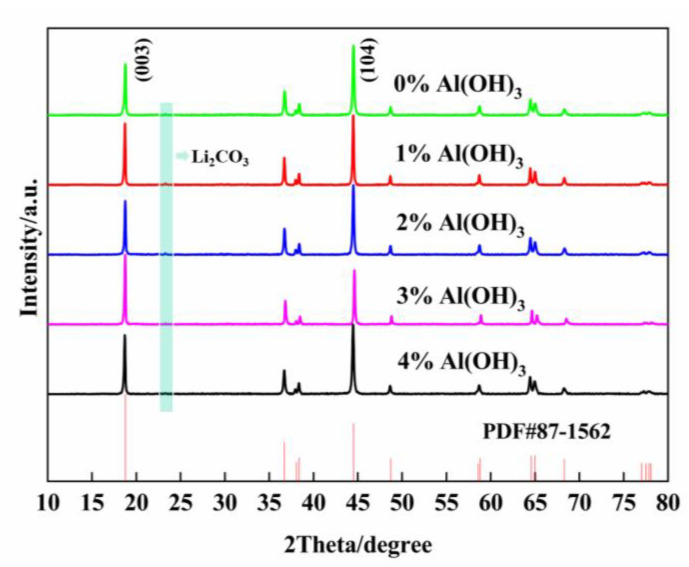
XRD patterns of regenerated cathode materials with different contents of added Al(OH)_3_.

Considering that the Al_2_O_3_ coating layers may have effects on the structure of the regenerated cathode material, XRD analysis of the regenerated cathode material was carried out, and the results are presented in Figure 2 and Table 2. The (003) XRD peaks are correlated with hexagonal structures, while the combination of cubic and hexagonal structures can be reflected by the (104) XRD peaks. The intensity ratio of peak (003) to peak (104) is closely related to the mixing state of Ni^2+^ and Li^+^ [31], and its theoretical value is greater than 1.2. The intensity ratio I_(003)_/I_(104)_ greater than 1.2 indicates a low degree of cation mixing. When I_(003)_/I_(104)_ > 1.3, it indicates a high degree of structural order in the material. Figure 2 illustrates that all the XRD patterns of the regenerated cathode materials are consistent with those of the standard PDF card, PDF#87-1562. There is a small impurity peak near 24°,which is inferred to be Li_2_CO_3_ (PDF#00-001-0996). When the content of added Al(OH)_3_ was less than 5%, peaks of Al_2_O_3_ were not present. Table 2 lists the lattice constants of LiNi_0.8_Co_0.15_Al_0.05_O_2_ with different contents of added Al(OH)_3_. Table 2 summarizes that with the increase in the added amount of Al(OH)_3_, the intensity ratio I_(003)_/I_(104)_ first increases and then decreases. When the added amount of Al(OH)_3_ is 3%, the intensity ratio I_(003)_/I_(104)_ is 1.485, which is greater than 1.3. Thus, this regenerated cathode material shows the lowest degree of cation mixing, the highest structural order, and the most stable structure. The material can be expressed as LiNi_0.8_Co_0.15−x_Al_x_O_2_@1.5%Al_2_O_3_.

#### 2.1.3. SEM and EDS Analyses

**Figure 3:**
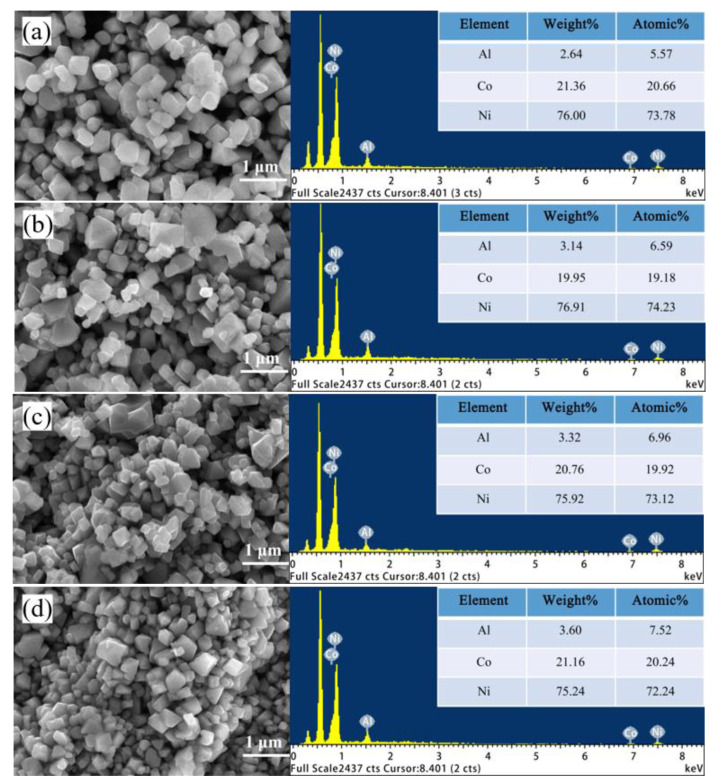
SEM images and EDS-mapping of the regenerated cathode materials with different contents of added Al(OH)_3_: ((**a**): 1%, (**b**): 2%, (**c**): 3%, and (**d**): 4%).

Figure 3 shows the morphology and energy spectrum of the regenerated cathode materials. As the pristine LiNi_0.8_Co_0.15_Al_0.05_ is already synthesized in our previous work [29], clearly, the actual content of Al element in the Al_2_O_3_ coated LiNi_0.8_Co_0.15_Al_0.05_ increases with the increasing amount of Al (OH)_3_ added according to the EDX surface composition analysis, while Co and Ni show no significant increase or decrease but remain stable at about 20 ato.% and 73 ato.%, respectively. Therefore, it is speculated that there is a rich Al phase on the surface. Notably, as Al_2_O_3_ can be obtained by the decomposition of Al(OH)_3_ at a low temperature of 140–150 °C, it is inferred that the rich Al phase may be in the form of an Al_2_O_3_ layer. When the added contents of Al(OH)_3_ are 1% and 2%, respectively, the particle distribution is relatively uniform. When the added content is between 3% and 4%, the surface morphology of the anode material is agglomerated. Consequently, the amount of Al in the material has a direct influence on the agglomeration of morphology, which further affects the electrochemical performance of the material.

#### 2.1.4. TEM Analysis

**Figure 4:**
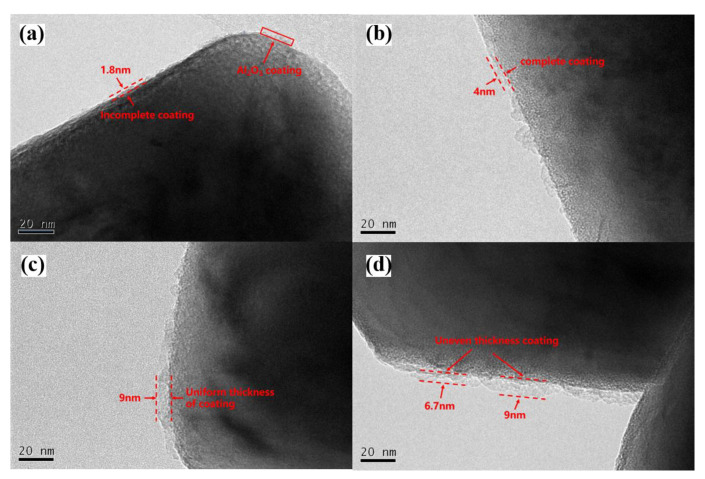
TEM images of the regenerated cathode materials with different added contents of Al(OH)_3_ addition: ((**a**): 1%, (**b**): 2%, (**c**): 3%, and (**d**): 4%).

The TEM analysis was conducted for the regenerated cathode material to determine whether an Al_2_O_3_ layer existed on its surface, and the corresponding results are shown in Figure 4. The surface of the regenerated cathode material is observed to be coated with a layer, and the thickness of the layer gradually increases with the addition of Al(OH)_3_. When the added amount of Al(OH)_3_ is 1%, the surface is not completely covered by the layer. In contrast, when the amount is 2%, the coating area increases, and the surface can basically be covered. Further, when the added amount is increased to 3%, the thickness of the coating becomes relatively uniform and the surface gets covered thoroughly. With the further increase in the added amount to 4%, the thickness of the surface coating layer increases significantly; however, the coating layer becomes uneven. Therefore, it is considered that the regenerated cathode material with 3% Al(OH)_3_ is the best, and accordingly, the material is denoted as LiNi_0.8_Co_0.15_Al_0.05_O_2_@1.5%Al_2_O_3._

#### 2.1.5. XPS Analyses

**Figure 5:**
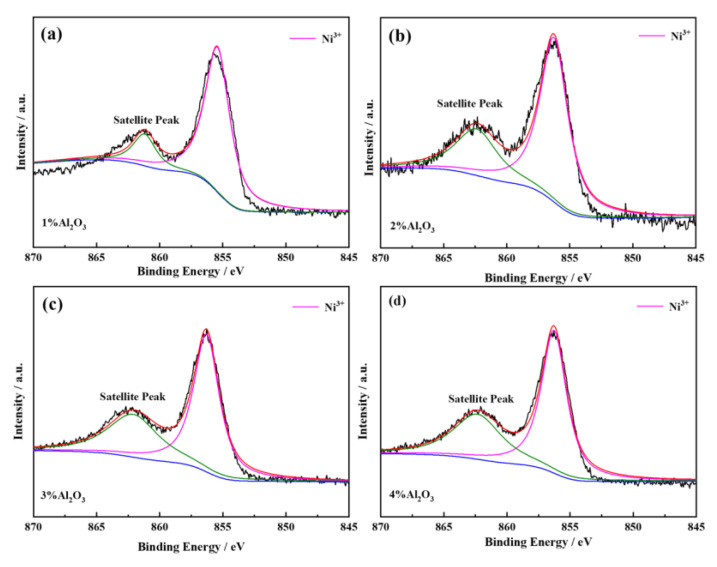
XPS spectra and corresponding fitting curves of the regenerated cathode materials with different amounts of added Al(OH)_3_: ((**a**): 1%, (**b**): 2%, (**c**): 3%, and (**d**): 4%).

In order to further determine the effect of different contents of added Al(OH)_3_ on the valence state of Ni ions in the regenerated cathode material, XPS tests were conducted. During the synthesis of this type of material with high Ni content, although it is expected that all Ni exists in the form of Ni^3+^, it is difficult to avoid the generation of low-priced Ni^2+^. The residual Ni^2+^ in LiNi_0.8_Co_0.15_Al_0.05_O_2_ inevitably occupies the position of Ni^3+^. Therefore, the cationic charge is reduced. In order to maintain the charge balance, part of Ni^2+^ migrates into the position of Li^+^. Then the cation mixing of Ni^2+^ and Li^+^ occurred [32]. The radius of Ni^2+^ (0.70 Å) is smaller than that of Li^+^ (0.74 Å), and it gets oxidized to Ni^3+^ (0.56 Å) during the process of delithiation, resulting in the collapse of the local hexagonal layered structure, thus making it difficult for lithium reintercalation. This will eventually make the thermal stability of the material worse and cause a high first irreversible discharge capacity. Figure 5 shows the XPS analysis results of the regenerated cathode materials with different amounts of added Al(OH)_3_. The content of Ni^3+^ in the regenerated material with Al_2_O_3_ coating is higher, and no Ni^2+^ is detected, which indicates that the presence of Al_2_O_3_ coating can inhibit the formation of Ni^2+^ and promote the formation of Ni^3+^. The Al_2_O_3_ coating has a positive effect on the material’s structure and performance.

#### 2.1.6. IR Analyses

**Figure 6:**
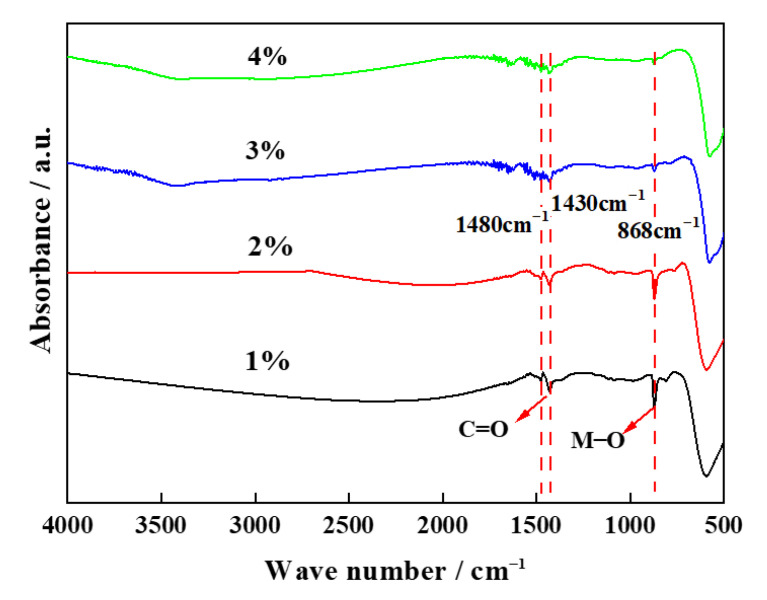
IR analysis of regenerated cathode material with coating.

IR analysis was conducted to evaluate the chemical bonds of regenerated cathode materials with different amounts of Al(OH)_3,_ and the corresponding results are shown in Figure 6. A strong peak is observed at 500–1000 cm^−1^, which corresponds to the M–O bond in MO_x_ (M = Ni, Co, Al). This result indicates that an oxide of Ni, Co, or Al was formed. Moreover, the intensity of the peak at 500–1000 cm^−1^ gradually decreases with the increase in the added amount of Al, which is due to the decrease in the percentage of M–O bonds caused by the increase in the percentage of the Al–O bonds. It also indicates the presence of Al_2_O_3_ within the material or on its surface. Two observed peaks at 1000–2000 cm^−1^ corresponds to C=O of CO_3_^2−^, indicating that the sample will contain traces of Li_2_CO_3_, which is consistent with the XRD result. Figure 6 clearly demonstrates that the intensity of the peak for the C=O group decreases for an Al content of 3% or 4%, which is possibly due to the decrease in the residual Li_2_CO_3_. Therefore, the addition of Al(OH)_3_ promotes the reaction and leads to a more adequate reaction of Li_2_CO_3_, which is beneficial for the synthesis of regenerated cathode materials.

The abovementioned analysis indicates that when the addition of Al(OH)_3_ is 3%, the regenerated cathode material LiNi_0.8_Co_0.15_Al_0.05_O_2_@1.5%Al_2_O_3_ exhibits the best performance.

### 2.2. Electrochemical Tests

#### 2.2.1. Initial Charge and Discharge Tests

**Figure 7:**
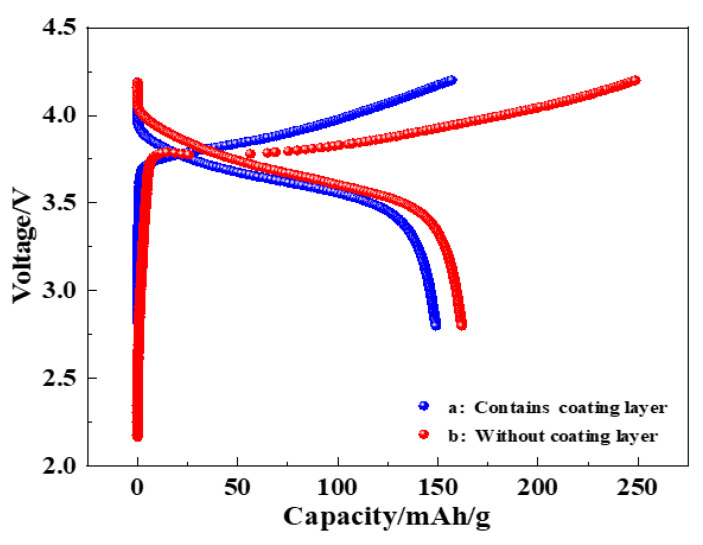
Initial charge–discharge test: (a: LiNi_0.8_Co_0.15_Al_0.05_O_2_@1.5%Al_2_O_3_ battery and b: LiNi_0.8_Co_0.15_Al_0.05_O_2_ battery).

The initial charge–discharge performance of the button batteries with the regenerated LiNi_0.8_Co_0.15_Al_0.05_O_2_@1.5%Al_2_O_3_ and the regenerated LiNi_0.8_Co_0.15_Al_0.05_O_2_ as cathode materials was investigated, as shown in Figure 7. The first charge capacity was 154 mA·h g^−1^, and the first discharge capacity was 150 mA·h g^−1^ for the LiNi_0.8_Co_0.15_Al_0.05_O_2_@1.5%Al_2_O_3_ battery. Therefore, the initial charge–discharge efficiency was 97.4% for the LiNi_0.8_Co_0.15_Al_0.05_O_2_@1.5%Al_2_O_3_ battery. In contrast, the initial charge specific capacity was 248.7 mA·h g^−1^, the initial discharge specific capacity was 162 mA·h g^−1^, and the initial charge–discharge efficiency was 65.1% for LiNi_0.8_Co_0.15_Al_0.05_O_2_ battery. It can be inferred that, through the coating of Al_2_O_3_, the initial charge–discharge efficiency of the battery increased by nearly 50%.

#### 2.2.2. Electrochemical Impedance Spectroscopy Tests

**Figure 8:**
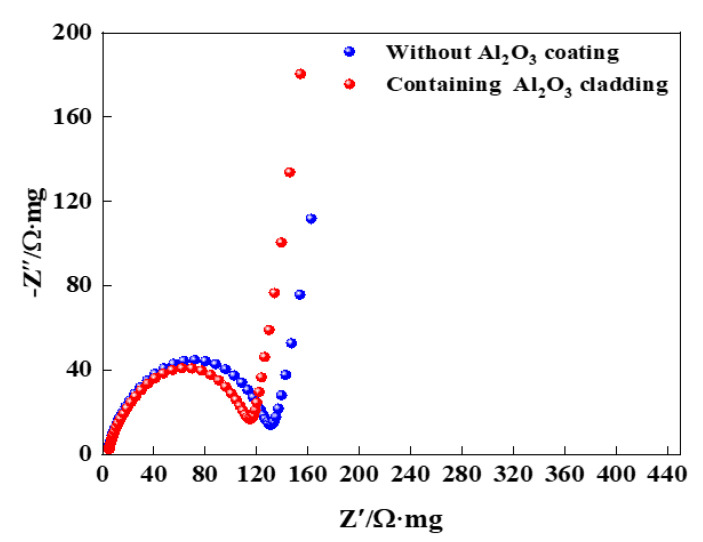
Impedance spectrum: (bule: LiNi_0.8_Co_0.15_Al_0.05_O_2_ battery and red: LiNi_0.8_Co_0.15_Al_0.05_O_2_@1.5%Al_2_O_3_ battery).

**Figure 9:**
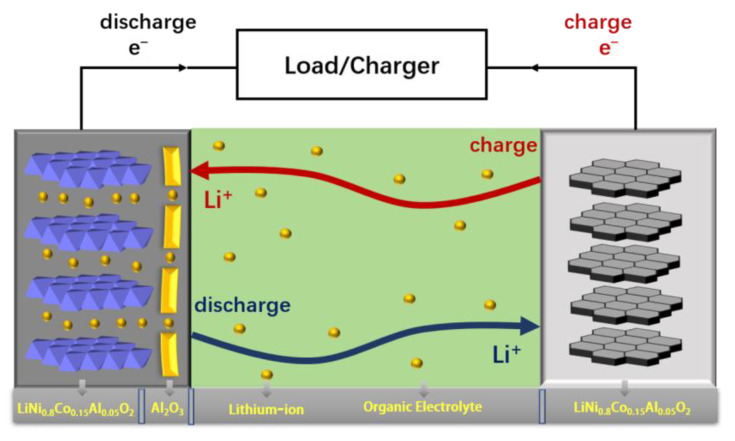
Schematic showing charge–discharge process of regenerated LiNi_0.8_Co_0.15_Al_0.05_O_2_@1.5%Al_2_O_3_.

Electrochemical impedance spectroscopy (EIS) tests were performed on the battery assembled with regenerated LiNi_0.8_Co_0.15_Al_0.05_O_2_@1.5%Al_2_O_3_ and regenerated LiNi_0.8_Co_0.15_Al_0.05_O_2_ as cathode material, as shown in Figure 8. EIS plots consist of a semicircle in the high frequency region associated to the solution resistance (Rs) and another semicircle in the low frequency region corresponding to the charge transfer resistance (Rct) [33,34]. The interface impedance of the LiNi_0.8_Co_0.15_Al_0.05_O_2_ battery corresponding to the semicircle in the high frequency region is 130 Ω, while that of the LiNi_0.8_Co_0.15_Al_0.05_O_2_@1.5%Al_2_O_3_ battery is 116 Ω. It possibly indicates that the Al_2_O_3_ coating can reduce the interfacial resistance. The slope of the low-frequency linear part for the LiNi_0.8_Co_0.15_Al_0.05_O_2_@1.5%Al_2_O_3_ battery is slightly larger than that of the LiNi_0.8_Co_0.15_Al_0.05_O_2_ battery, indicating that the diffusion impedance of the LiNi_0.8_Co_0.15_Al_0.05_O_2_@1.5%Al_2_O_3_ battery is lower. The Rct of the Al_2_O_3_ coated sample has a smaller Rct, potentially indicating a better ion-conducting phase formed, which effectively reduces undesirable phase changes, buffers the inherent stresses and strains between the binder, cathode, and current collector, and avoids volume changes, thus increasing the conductivity [35]. In addition, when the pristine cathode material is exposed to air, the residual lithium carbonate promotes Ni^3+^ reduction to Ni^2+^ to form the Ni-O layer. The residual lithium carbonate and Ni-O layer not only hinder the diffusion of Li^+^ and charge transfer at the interface but also accelerate the decomposition of electrolyte to produce corrosive substances such as HF [36]. This situation can be improved by the Al_2_O_3_ layer, which in turn improves the electrochemical performance of the pristine cathode material. The structure and the charge–discharge process for a LiNi_0.8_Co_0.15_Al_0.05_O_2_@1.5%Al_2_O_3_ battery are shown in Figure 9.

#### 2.2.3. Electrochemical Performance Analysis

**Figure 10:**
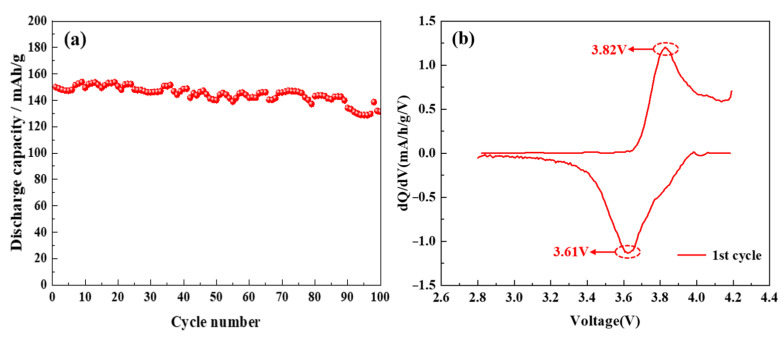
Electrochemical performance: ((**a**): Cycle number-specific capacity curve and (**b**): dQ/dV curve).

Furthermore, the electrochemical analysis of the LiNi_0.8_Co_0.15_Al_0.05_O_2_@1.5%Al_2_O_3_ battery was carried out. Figure 10a shows the relationship between the number of cycles and discharge specific capacity. The discharge specific capacity is 140.1 mA·h g^−1^ at 0.5C after 50 cycles, and the capacity retention rate is 93.4%. However, the discharge specific capacity and capacity retention rate are 131.4 mAHg^−1^, or 87.6%, after 100 cycles. This also initially shows the performance of the battery [37,38]. Figure 10b demonstrates the dQ/dV curve, which shows only one distinct redox couple of an oxidation peak and a reduction peak in the voltage range of 2.5–4.5 V. The results show that the crystal structure of the material does not change, so Li^+^ has good intercalation and deintercalation abilities [39,40]. Consequently, the LiNi_0.8_Co_0.15_Al_0.05_O_2_@1.5%Al_2_O_3_ battery exhibits good cycling and structural stability.

## 3. Experimental

### 3.1. Recycling of Cathode Materials

**Figure 11:**
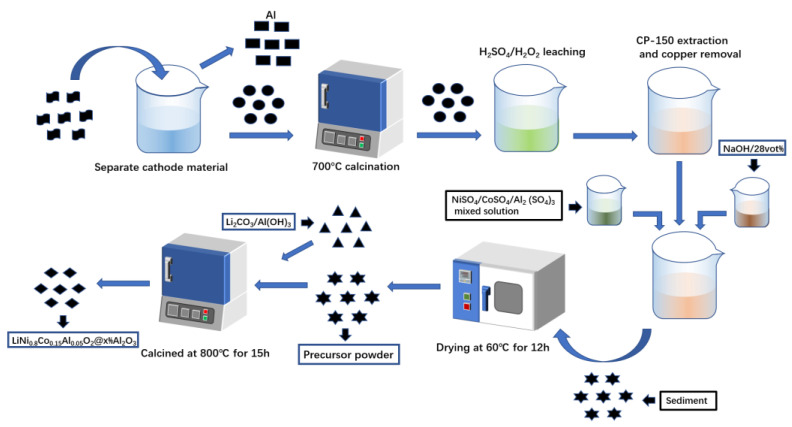
Flow chart of the regeneration process.

The spent battery cathode material was peeled from the positive plate using a NaOH solution. High-temperature pyrolysis of spent cathode materials at 700 °C was followed by acid-leaching with H_2_SO_4_ (2 mol L^−1^) and H_2_O_2_ (10% by volume) at 90 °C for 2 h. The leaching solution containing 4.9 g L^−1^ of Co, 25.2 g L^−1^ of Ni, 0.21 g L^−1^ of Cu, and 0.68 g L^−1^ of Al was obtained. CP-150 was used to remove the Cu^2+^ impurity from the acid leaching solution. The optimal extraction conditions were determined as: oil phase/aqueous phase (O/A) 2:1, pH 3, and an extractant concentration of 30%. Appropriate amounts of CoSO_4_, NiSO_4,_ and Al_2_(SO_4_)_3_ with a n(Ni): n(Co): n(Al) ratio of 80:15:5 were added to the raffinate. 6 mol L^−1^ of NaOH solution was thoroughly mixed with 28 vol% of NH_3_·H_2_O to obtain a mixed solution. The pH of the raffinate solution was adjusted to 10.7 at 50 °C for 1.5 h to obtain the precipitate. The drying condition of the precipitate is as follows: dry at 60 °C for 12 h, then grind it into powder containing Co, Ni, and Al, which was used as the precursor powder. The preparation procedure for this precursor powder was reported in our previous study [29]. Then the precursor powder was mixed thoroughly with Li_2_CO_3_ and Al (OH)_3_, and then calcined in a muffle furnace to obtain the regenerated cathode material, LiNi_0.8_Co_0.15_Al_0.05_O_2_@x%Al_2_O_3_. The addition amounts of n (precursor): n (Li_2_CO_3_) were 1:1.1. The precursor and Al (OH)_3_ were in different proportions of 1:1%, 1:2%, 1:3%, and 1:4%, respectively. The calcination temperature was 800 °C and the calcination time was 15 h. The entire process is depicted in Figure 11.

### 3.2. Characterization

The structures of the regenerated cathode materials were investigated by XRD (D8-Advance, Bruker, Germany). The morphologies and detailed structures of the cathode material were characterized by SEM (JSM-5600LV, JEOL, Japan, conditions: operating voltage 20 kV, amplification factor 100,000 times) and Transmission electron microscope (TEM, JEOL JEM-2010). Thermogravimetric analysis (TGA, Labsysevo, France, conditions: 0–1000 °C, 10 °C min^−1^) was used to analyze the thermal stability of the cathode material. A Fourier transform infrared spectrometer (FTIR-650, Guangdong FTIR-650) was used to analyze the cathode material in mid-infrared mode. The X-ray photoelectron spectroscope (XPS, Sigma Probe X-ray, THERMO VG, UK) was used to characterize the chemical states of the cathode material using C 1s (284.6 eV) as the calibration reference.

### 3.3. Electrochemical Tests

The preparation and testing of the electrochemical properties of button cells using regenerated materials involve the use of acetylene black and polyvinylidene difluoride (PVDF) that were mixed with a weight ratio of 8:1:1 and added into a small beaker, and a moderate amount of N-methyl pyrrolidone (NMP) was added to this mixture and mixed thoroughly. Then the slurry was coated on the Al foil and was dried in a vacuum drying oven at 70 °C. Lithium metal was used as the counter electrode of this half-cell, and 1 M LiPF6 (ethylene carbonate (EC): dimethyl carbonate (DMC): ethyl methyl carbonate (EMC) = 1:1:1, *v*/*v*)) was used as the electrolyte, and the batteries were assembled in an argon-filled glove box (Etelux). Galvanostatic cycling tests were carried out on the LAND CT2001A battery tester (conditions: voltage 2.5–4.3 V versus Li^+^/Li, current density: 0.2C, (1C = 278 mA g^−1^)). Electrochemical impedance spectroscopy (EIS) was tested by an electrolytic workstation (Autolab, 100–0.01 Hz).

## 4. Conclusions

The regenerated coated cathode material, i.e., LiNi_0.8_Co_0.15_Al_0.05_O_2_@xAl_2_O_3_ was prepared in this study. TEM analysis showed that the addition of Al(OH)_3_ promoted the synthesis reaction. XRD analysis shows that the structure of the material attains a high degree of order. SEM and EDS analysis results reveal the existence of Al_2_O_3_ in the regenerated cathode material, which may be doped inside the material or coated on the surface. TEM analysis further confirms the formation of an Al_2_O_3_ coating, and the thickness of the coating increases with the addition of Al(OH)_3_. XPS analysis shows that the existence of the Al_2_O_3_ coating can inhibit the formation of Ni^2+^ and help stabilize the structure. The regenerated cathode material with 3% Al(OH)_3_, i.e., LiNi_0.8_Co_0.15_Al_0.05_O_2_@1.5%Al_2_O_3_ shows the best performance in all aspects. Compared with a LiNi_0.8_Co_0.15_Al_0.05_O_2_ battery, the first charge–discharge efficiency of the battery assembled with LiNi_0.8_Co_0.15_Al_0.05_O_2_@1.5%Al_2_O_3_ increases to 97.4%, and the interface impedance and diffusion impedance are lower. The capacity retention rate of the LiNi_0.8_Co_0.15_Al_0.05_O_2_@1.5%Al_2_O_3_ battery is 93.4% after 50 cycles and 87.6% after 100 cycles, thus indicating its good cycling and structural stability. This work provides a simple and effective method for the regeneration of cathode materials for spent batteries.

## Figures and Tables

**Table 1:** Processes of foreign battery recycling companies [19,20,21].

Craftsmanship	Battery Type	Other Products
Hydrometallurgy and electrochemistry	LiOH	Co_2_O_3_, Al, Cu etc.
Spent batteries Restoration	LiCoO_3_	Electrode materials Cu, Al
Low-temperature ball milling	Li_2_CO_3_	Co, Ni
Hydrometallurgy	Li_2_CO_3_ or Li_3_PO_4_	Cu
Pyrometallurgy	——	CoCl_2_, Cu

**Table 2:** Lattice constants of regenerated cathode materials with different contents of added Al(OH)_3_.

Peak (003) Intensity	Peak (104) Intensity	Added Amount of Al(OH)_3_ (%)	Intensity Ratio I_(003)_/I_(104)_
10,631	12,706	0	0.836
12,035	13,686	1	0.879
16,688	15,930	2	1.047
19,327	13,013	3	1.485
10,837	11,355	4	0.954

## Data Availability

Not applicable.

## Sample Availability

Samples of the compounds are available from the authors.
